# Exploring the Potential of β-Catenin *O*-GlcNAcylation by Using Fluorescence-Based Engineering and Imaging

**DOI:** 10.3390/molecules25194501

**Published:** 2020-10-01

**Authors:** Angelina Kasprowicz, Corentin Spriet, Christine Terryn, Vincent Rigolot, Stephan Hardiville, Matthew G. Alteen, Tony Lefebvre, Christophe Biot

**Affiliations:** 1 Univ. Lille, CNRS, UMR 8576–UGSF–Unité de Glycobiologie Structurale et Fonctionnelle, F-59000 Lille, France; angelina.kasprowicz@univ-lille.fr (A.K.); corentin.spriet@univ-lille.fr (C.S.); vincent.rigolot.etu@univ-lille.fr (V.R.); stephan.hardiville@univ-lille.fr (S.H.); tony.lefebvre@univ-lille.fr (T.L.); 2 Univ. Lille, CNRS, Inserm, CHU Lille, Institut Pasteur de Lille, US 41–UMS 2014–PLBS, F-59000 Lille, France; 3 PICT Platform, University of Reims Champagne-Ardenne, 51 rue Cognacq-Jay, 51100 Reims, France; christine.terryn@univ-reims.fr; 4 Department of Chemistry, Simon Fraser University, Burnaby, BC V5A 1S6, Canada; malteen@sfu.ca

**Keywords:** bioorthogonal chemistry, fluorescence, glycosylation, metabolic incorporation, GFP, β-catenin

## Abstract

Monitoring glycosylation changes within cells upon response to stimuli remains challenging because of the complexity of this large family of post-translational modifications (PTMs). We developed an original tool, enabling labeling and visualization of the cell cycle key-regulator β-catenin in its *O*-GlcNAcylated form, based on intramolecular Förster resonance energy transfer (FRET) technology in cells. We opted for a bioorthogonal chemical reporter strategy based on the dual-labeling of β-catenin with a green fluorescent protein (GFP) for protein sequence combined with a chemically-clicked imaging probe for PTM, resulting in a fast and easy to monitor qualitative FRET assay. We validated this technology by imaging the *O*-GlcNAcylation status of β-catenin in HeLa cells. The changes in *O*-GlcNAcylation of β-catenin were varied by perturbing global cellular *O*-GlcNAc levels with the inhibitors of *O*-GlcNAc transferase (OGT) and *O*-GlcNAcase (OGA). Finally, we provided a flowchart demonstrating how this technology is transposable to any kind of glycosylation.

## 1. Introduction

The complete set of glycans, i.e., the glycome, represents a large diversity of structures and functions and plays a central role in biology and health [1]. Glycans are involved in many biological processes, such as protein folding and trafficking, cell-to-cell recognition, immune defense, and cell protection [1]. Glycans are also specific markers for numerous pathologies, including cancers [2]. This functional diversity is linked to a great structural diversity of glycans. Indeed, a wide variety of different forms of glycosylation are possible, and, moreover, variation in glycosylation of the same protein within tissues can occur, leading to various “glycoforms” of a given protein [3].

The various forms of glycosylation are essential for cell homeostasis. In this regard, it is estimated that 2% of the human genome is devoted to glycosylation. The most complex forms of glycosylation, namely *N*-linked protein, *O*-linked protein, and *O*-linked mucin-type glycosylation, take place mainly in the endoplasmic *reticulum* and the Golgi apparatus where the requisite biosynthetic enzymes reside. Another widespread form of glycosylation is *O*-linked β-*N*-acetylglucosaminylation (*O*-GlcNAcylation) [4]. *O*-GlcNAcylation is much simpler than glycosylation occurring within the secretory pathway and consists of the modification of cytoplasmic, nuclear, and mitochondrial proteins with a single residue of *N*-acetylglucosamine (GlcNAc). Furthermore, in contrast to the more complex glycosylation of the secretory pathway, *O*-GlcNAcylation is highly dynamic, its versatility being managed by a unique pair of enzymes—*O*-GlcNAc transferase (OGT) and *O*-GlcNAcase (OGA)—which, respectively, install and remove the GlcNAc moiety on and off targeted substrate proteins [5]. *O*-GlcNAcylation can compete with phosphorylation at the same or at neighboring sites, leading to a reciprocal interplay between the two post-translational modifications (PTMs) [4,6].

During the last three decades, metabolic oligosaccharide engineering (MOE, also termed metabolic glycoengineering (MGE)) has paved the way for the manipulation of biosynthetic pathways responsible for oligosaccharide and glycoconjugate production [7,8,9,10]. MOE exploits the substrate promiscuity of different glycosylation pathways to incorporate non-natural monosaccharides into cellular glycans. Upon metabolization, the non-natural saccharides are detected with a complementary probe through bioorthogonal ligation. This technology has been mostly used for labeling cell surface-exposed glycoconjugates, especially with fluorescent probes [7,8], but there are some intracellular MOE applications, notably targeting *O*-GlcNAc.

However, these strategies usually aim to report the general glycosylation status of a cell, and, to date, only a few MOE methods have been reported that account for the glycosylation pattern of a single specific glycoprotein. The five examples are based on dual-labeling strategies, exploiting Förster resonance energy transfer (FRET). Haga and coworkers used azido sialic acid (SiaNAz) labeling of GLUT4-GFP to image cell surface glycoproteins [3]. Belardi and coworkers developed a technique using Ac_4_ManNAz (the precursor of SiaNAz) to study the vitronectin receptor α_V_β3 after targeting with a donor fluorophore-labeled Fab fragment [11]. The dual-labeling of membrane proteins developed by Lin and coworkers relies on two bioorthogonal chemical reporters delivered to both the protein and the glycan [12]. Surprisingly, only modest progress has been made in studying intracellular *O*-GlcNAcylation. The two examples to visualize *O*-GlcNAcylation in a protein-specific manner have been reported by Lin and coworkers [13] and Doll and coworkers [14].

Herein, we presented optimized methods for probing the glycosylation state of β-catenin within HeLa cells using fluorescence-based imaging. β-catenin plays many critical functions in animals. First, Wnt/β-catenin signaling—for which the protein is the central component—is actively involved in embryonic development from flies to mammals [15]. β-catenin is also crucial for cell cycle progression and for the maintenance of the adherens junctions in epithelia. β-catenin is a proto-oncoprotein, its upregulation being responsible for tumorigenesis in a wide variety of cancers. The fate of the protein is well established as being controlled by phosphorylation of a D-box located at the *N*-termini: phosphorylation at S33, S37, T41, and S45 triggers the poly-ubiquitination of β-catenin and its subsequent targeting to the 26S proteasome [16]. Therefore, mutations of the D-box or deletions of the N-termini of β-catenin are frequently found in patient tumors. We previously demonstrated that the *O*-GlcNAcylation of β-catenin reduced its susceptibility to proteasomal degradation. In particular, the modification of T41 with *O*-GlcNAc was found to interfere with the sequential phosphorylation and ubiquitinylation, which led to the destruction of this protein [6]. Given these important biological roles and defined functions for specific post-translational modifications, β-catenin appeared to us as the model of choice for the development of our strategy to visualize specific glycosylated proteins in cells.

## 2. Results

Among the in-situ visualization techniques, the use of GFP and its derivatives is the gold standard to study genetically encoded biomolecules, such as proteins. However, for all other non-genetically encoded biomolecules, such as glycans or lipids, GFP-based imaging techniques are not feasible. Therefore, it is necessary to exploit novel techniques to enable visualization of these compounds.

**Figure 1:**
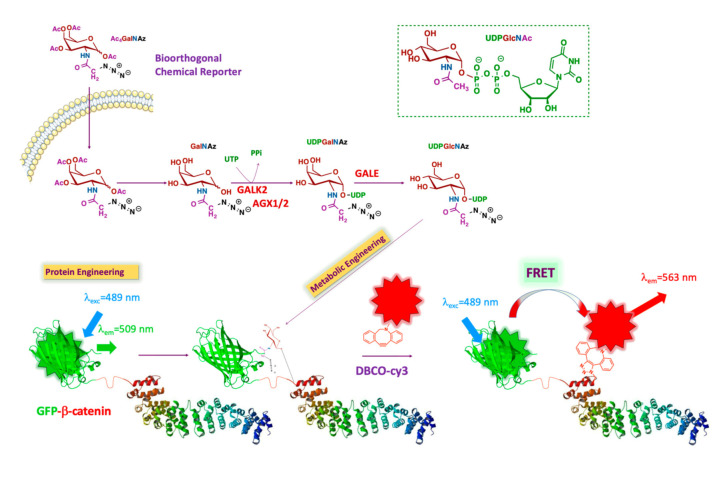
Schematic representation of the strategy: FRET-based imaging of dual-labeling of protein and glycans. FRET, Förster resonance energy transfer.

We opted for a bioorthogonal chemical reporter strategy to detect the *O*-GlcNAc modification state of β-catenin. This technology is based on the incorporation of a chemically-modified metabolic precursor of UDP-GlcNAc [17], bearing a pendant bioorthogonal handle at the 2-position of the pyranose ring. This chemically reactive analog permits conjugation with a fluorophore to generate an imaging probe. This approach allows the in vivo study of *O*-GlcNAc-modified β-catenin without interfering with native biological processes (Figure 1). The technology we proposed involves the dual-labeling of β-catenin with a genetically encoded GFP tag, followed by the bioorthogonal installation of a second fluorophore on the *O*-GlcNAc unit. The combined use of two fluorescent reporters in close proximity to each other enables a convenient and efficient FRET assay suitable for imaging cells.

β-catenin is genetically labeled with GFP (donor). The intracellular *O*-GlcNAcylated proteins are labeled by metabolic oligosaccharide engineering (MOE), followed by bioorthogonal chemistry with a fluorophore (acceptor). As the distance between donor and acceptor is less than 10 nm, the only acceptor bound to the glycans on β-catenin is excited via intramolecular FRET. The β-catenin structure is adapted from Huber et al. [17].

Briefly, the chemical structure of UDP-GlcNAc is shown in the upper right in Figure 1 (green box). The different colors represent the various metabolic origins of the nucleotide-sugar, as follows: brown, carbohydrate metabolism; blue, glutamine metabolism; purple, metabolism of fatty acids and ketogenic amino acids; green, nucleotide metabolism. The unnatural GlcNAc analog used for our tracing strategy was Ac_4_GalNAz (see below). This reporter enters the cell by passive diffusion. Thereafter, Ac_4_GalNAz is deacetylated by esterases and then further metabolized to generate UDP-GalNAz. After isomerization into UDP-GlcNAz by the epimerase GALE (UDP-glucose 4-epimerase), OGT transfers GlcNAz to a set of proteins, including GFP-β-catenin. The addition of a compatible alkyne-labeled fluorophore results in the conjugation of the fluorophore to the azide handle of the GlcNAz unit through a strain-promoted azide-alkyne cycloaddition (SPAAC). This permits FRET between the GFP group and the fluorophore conjugated to the sugar, making it possible to specifically visualize *O*-GlcNAcylated β-catenin in cells.

### 2.1. Optimization of Fusion Protein Linker

**Figure 2:**
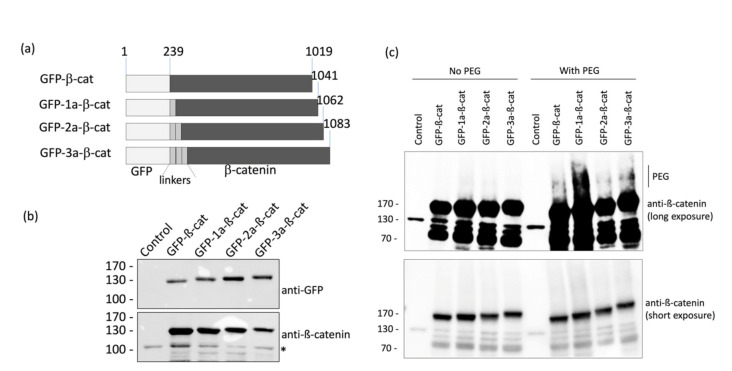
Design and validation of fluorescent β-catenin fusion proteins. (**a**) Schematic representation of the different GFP-β-catenin constructs used in this study. (**b**) HeLa cells were transfected with the various GFP-β-catenin expressing vectors. Cell lysates were analyzed by western blots using anti-GFP and anti-β-catenin antibodies. The asterisk indicates the detection of the endogenous form of β-catenin. (**c**) Protein extracts of HeLa cells were labeled with 4.4 kDa DBCO-PEG mass tag or incubated with DMSO. β-catenin proteins were detected by Western blot.

The linker between β-catenin and GFP is an important consideration in order to satisfy the requirements for FRET, including the distance between fluorophore pairs (<10 nm) and dipole orientation. Therefore, four different fusion proteins were investigated with either no linker or 1-to-3 α-helix linkers (1a, 2a, and 3a) inserted between the GFP and the β-catenin domains: (1) GFP-β-catenin, (2) GFP-1a-β-catenin, (3) GFP-2a-β-catenin, and (4) GFP-3a-β-catenin (Figure 2a). As a lack of rigidity of these linkers can be a limitation for FRET-based systems [18], a semi-flexible peptide linker (EAAKEAAAKEAAAKEAAAKA)_1-3_, which adopts an alpha-helix conformation, was chosen to bridge the domains. We envisioned this linker would strike a balance in the degree of mobility between the two domains, maximizing the potential for efficient FRET.

The different N-terminal GFP-tagged β-catenins were produced in HeLa cells and analyzed by Western blotting in order to assess the expression level of each form (Figure 2b). We found that all four fusion protein constructs were suitably expressed and, therefore, chose to assess each variant in microscopy experiments. Note that the insertion of linkers increased the apparent molecular weight of GFP-β-catenin depending on the number repeats between the two domains.

In order to ascertain that the different GFP-β-catenin constructs were modified following the incubation of HeLa cells with Ac_4_GalNAz, a mass tag labeling was performed, as described in the materials and methods session. As shown in Figure 2c, we observed that the different constructs were PEGylated, as indicated by the shift in the upper molecular weight when compared to the control condition (no PEG). This experiment asserted that the GFP-β-catenin constructs were labeled with the GlcNAz moiety and then clicked by the DBCO-PEG.

**Figure 3:**
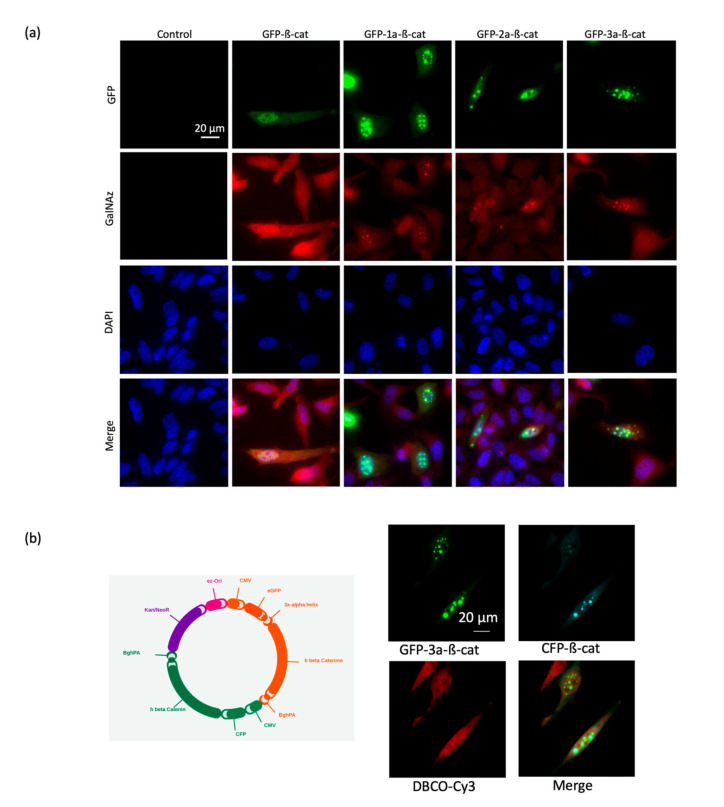
Evaluation of the GFP-β-catenin constructs via fluorescent microscopy. (**a**) Fluorescence images of HeLa cells expressing GFP-linker-β-catenins, treated with 200 µM Ac_4_GalNAz and labeled with DBCO-cy3. (**b**) Fluorescence images of HeLa cells expressing GFP-3a-β-catenin and CFP-β-catenin, treated with 200 µM Ac_4_GalNAz and labeled with 10 µM DBCO-cy3. The map of the vector encoding both the GFP-3a-β-catenin and the CFP-β-catenin is shown on the left. CFP, cyan fluorescent protein.

We next evaluated the four GFP-β-catenin constructs via fluorescent microscopy using fixed Hela cells (Figure 3). We found that all constructs produced acceptable levels of fluorescent signal and subcellular localization. To ensure that FRET will be related to the proximity between donor and acceptor and not to the relative orientation of each modification, we chose the most flexible construct (GFP-3a-β-catenin) for FRET experiments. Indeed, computational simulations showed that the orientation factor converged to a constant in a FRET sensor where the donor/acceptor pair was presumed to be freely mobile. Thus, the FRET signal of the chosen construct would be related only to the donor/acceptor distance [19].

Interestingly, a di-systronic expression vector was designed and produced in order to combine the direct synthesis and secretion of both the GFP-3a-β-catenin and a cyan fluorescent protein β-catenin construct (CFP-β-catenin). Fluorescence colocalization microscopy demonstrated that both proteins localized in the same subcellular compartments (Figure 3b). We chose to proceed with the GFP-β-catenin construct in future experiments of the improved spectral overlap of GFP emission with the excitation band of the Cy3 acceptor fluorophore. We also concluded that the length of the linker between the GFP and β-catenin domains had no dramatic effect on the integrity of the fusion protein.

### 2.2. Optimization of Metabolic Oligosaccharides Engineering (MOE)

The ligation reactions used in the metabolic oligosaccharides engineering (MOE) technology are based on the principle of “click chemistry“ introduced by the group of Sharpless [20]. A wide range of bioorthogonal reactions has been developed, including nucleophilic substitution reactions, carbonyl chemistry, and cycloaddition reactions between unsaturated compounds, such as Diels-Alder reactions or 1,3-dipolar cycloaddition reactions. Here, we chose the strain-promoted alkyne-azide cycloaddition (SPAAC) due to its ability to label glycans in live cells [21]. Being one of the most widely used cyclooctyne derivatives due to its robust stability and reactivity, we selected a fluorophore carrying a dibenzylcyclooctyne (DBCO), also known as ADIBO (azadibenzocyclooctyne) or DIBAC (dibenzoazacyclooctyne) [22].

As N-azidoacetylglucosamine (GlcNAz) turned out to be a weak metabolic labeling reagent [17], we instead used N-azidoacetylgalactosamine (GalNAz), which is more efficiently metabolized into UDP-N-GlcNAz in cells. In its peracetylated form (Ac_4_GalNAz), this derivative has the property of being cell-permeable.

**Figure 4:**
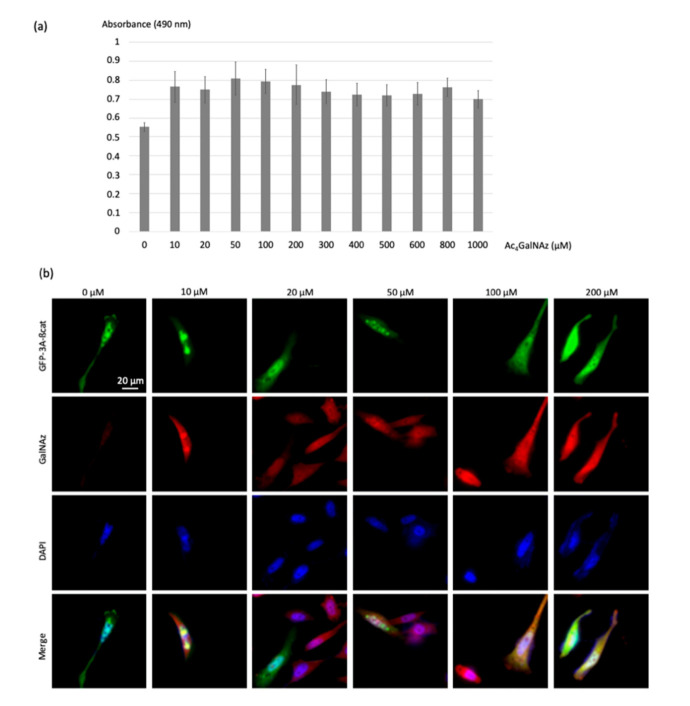
Optimization of the concentration of the chemical reporter. (**a**) The histogram represents the cytotoxicity evaluation of the chemical reporter Ac_4_GalNAz; Hela cells were treated at a concentration of 0 to 1000 µM. Twenty-four hours later, the cell viability was determined using the MTS assay. (**b**) HeLa cells transfected with the GFP-3a-β-catenin expressing vectors were treated with increasing Ac_4_GalNAz concentration (10, 20, 50, 100, and 200 µM) and labeled with DBCO-cy3 (10 µM).

To address the optimum Ac_4_GalNAz concentration for cell labeling and FRET analysis, HeLa cells were treated with increasing Ac_4_GalNAz concentration (10, 20, 50, 100 and 200, 300, 400, 500, 600, 800, 1000 µM) to introduce the O-GlcNAz onto intracellular O-GlcNAcylated proteins (Figure 4a). The viability of cells was then assessed using an MTS cell proliferation assay. No significant impact on proliferation was observed at concentrations up to 200 μM Ac_4_GalNAz, indicating that treatment did not induce cytotoxic effects.

The azidosugar was then labeled with the DBCO-cy3 FRET acceptor (10 µM) using the SPAAC reaction, permitting visualization of *O*-GlcNAz-modified β-catenin. Colocalization of green and red signals produced a yellow color, validating the dual-labeling (Figure 4b).

As shown in Figure 4b, no modification of the morphology of the cells was observed even at the highest dose of 200 µM of the chemical reporter, showing that the toxicity of the azidosugar concentration was negligible even at the highest concentrations. Acetic acid release inside cells upon enzymatic deacetylation of Ac_4_GalNAz did not seem to induce cytotoxicity, as was previously reported for Ac_4_ManNAz (>50 μM) [23]. In order to promote high FRET efficiencies, we chose the highest non-toxic Ac_4_GlcNAz concentration of 200 µM.

### 2.3. SLiM-FRET Readout Assay

**Figure 5:**
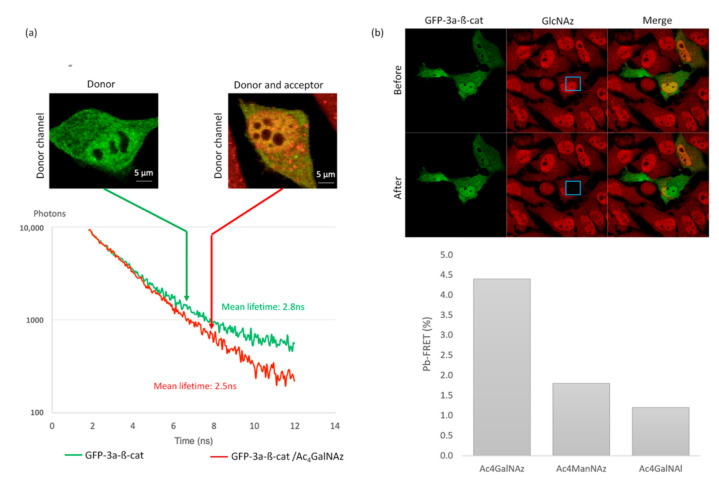
Proof of feasibility: FRET imaging of *O*-GlcNAcylated GFP-3a-β-catenin. (**a**) SLiM-FRET experiment using two-photon excitation: representative photon decay curves and associated fluorescence mean lifetime extracted from only the GFP emission channel. (**b**) Donor dequenching after acceptor photobleaching. The inserted blue box indicates the photobleached area. Histograms correspond to calculated pb-FRET inside the cells after photobleaching in comparison to Ac_4_GalNAl and Ac_4_ManNAz, which serve as a negative control.

To carry out a quantitative analysis of the FRET between the GFP and the cy3-labeled GlcNAc, a spectral fluorescence lifetime imaging microscopy (SLiM) acquisition system was used, thus acquiring, simultaneously, 16 photon decay curves along the fluorescence emission spectrum, and permitting unambiguous FRET measurements (Figure 5) [24]. In the absence of the chemical reporter, a mean fluorescence lifetime of 2.8 +/− 0.06 ns was observed in GFP-3a-β-catenin expressing cells (Figure 5a). We then imaged GFP-3a-β-catenin expressing cells that were treated with Ac_4_GalNAz and click-labeled with DBCO-cy3. Using these conditions, the mean fluorescence lifetime of the GFP reporter in Ac_4_GalNAz-treated cells decreased to 2.5 +/− 0.17 ns, which indicated that some of the GFP energy was transferred to the nearby cy3 fluorophore (Figure 5a). A FRET event could thus be observed with a *p*-value < 0.05.

These experiments allowed us to observe variations of up to 300 ps in lifetime experiments. This range corresponded to a deviation from the GFP-3a-β-catenin basal *O*-GlcNAcylation level to the non-*O*-GlcNAcylated state (Figure 5b). This dynamic range was sufficient to decipher subtle variations in *O*-GlcNAcylation levels.

We thus demonstrated that our GFP-3a-β-catenin GalNAz-cy3 SLiM-FRET strategy is sufficiently sensitive for use in live cells. This important point at this level of the study can be considered as a proof of concept for this new tool.

### 2.4. Pb-FRET Readout Assay

While SLiM allows quantitative and sensitive FRET measurements, it requires a dedicated acquisition system, and it is highly time-consuming. After validation of the biosensor using SLiM, we wanted to demonstrate the robustness of our method using a traditional confocal microscope to monitor FRET through a photobleaching FRET readout assay, a fast and widely available technology.

As depicted in Figure 5b, DBCO-cy3 was specifically photobleached within a defined region of the cell in order to keep an internal control [24]. Using pb-FRET, we observed a measurable FRET efficiency of 4.4% +/− 0.1% on GFP-3a-β-catenin expressing cells when treated with Ac_4_GalNAz and click-labeled with DBCO-cy3.

In order to confirm that measured FRET resulted from intramolecular FRET, we performed control experiments with GFP-3a-β-catenin and the alkyne-containing reporter Ac_4_GalNAl [19] (Figure 5b). This analog is unable to react with DBCO-cy3, allowing the evaluation of the possible contributions from intermolecular FRET between the two fluorophores. More than a thousand intracellular proteins are post-translationally modified with *O*-GlcNAc and thus susceptible to the incorporation of GlcNAz, followed by DBCO-cy3. However, due to the relatively low expression of GFP-β-catenin in our model system, we hypothesized that the concentration of donor and acceptor fluorophores would be low enough that contributions from intermolecular FRET would be negligible [25]. In support of this assumption, no measurable FRET compared to GFP-β-catenin alone was monitored, as confirmed by experiments presented in Figure 5b. Indeed, a low background signal of 1.2% +/− 0.5% was recorded when cells were treated with Ac_4_GalNAl.

Further, as control with another tagged monosaccharide (ManNAz), which cannot be metabolized into GlcNAz, we observed a low FRET efficiency of 1.8% +/− 0.7% on GFP-3a-β-catenin expressing cells when treated with Ac4ManNAz and click-labeled with DBCO-cy3 (Figure 5b). This is a typical example of the advantage of GalNAz labeling of β-catenin.

### 2.5. Pharmacology

**Figure 6:**
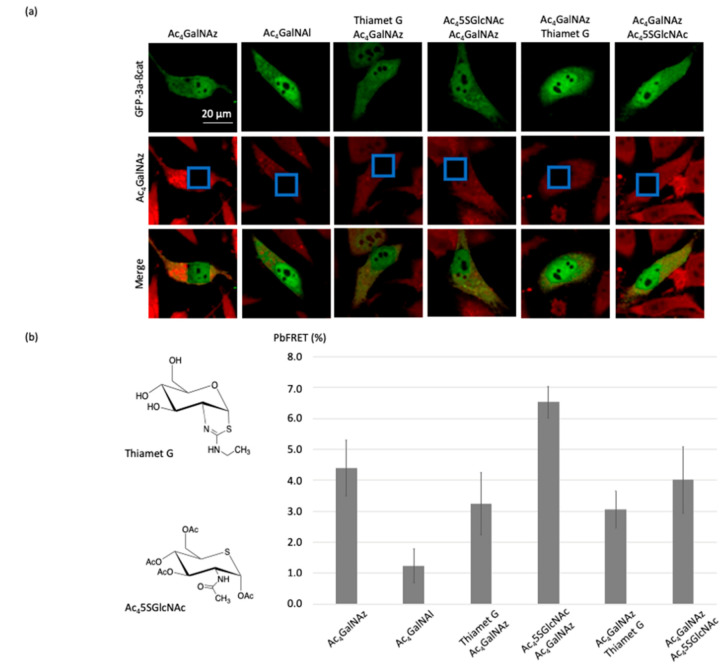
Imaging the changes of pb-FRET for GFP-3a-β-catenin in HeLa cells induced by the OGT and OGA inhibitors. (**a**) Fluorescence images of the *O*-GlcNAcylated glycoform of β-catenin before and after photobleaching using Thiamet-G or Ac_4_5SGlcNAc. (**b**) Histograms corresponding to calculated pb-FRET inside the cells after photobleaching using thiamet-G or Ac_4_5SGlcNAc, whose structures are shown at the left.

Having developed an efficient, robust, and easy to use the technique to process Hela cells for pb-FRET imaging, we used this approach to evaluate the pharmacological effects of thiamet-G and Ac_4_5SGlcNAc. Thiamet-G [26] and Ac_4_5SGlcNAc [16] are effective cell-active inhibitors of O-GlcNAcase (OGA) and O-GlcNAc transferase (OGT), respectively. OGT catalyzes the addition of O-GlcNAc to proteins [27]; OGA removes the modification [28]. Thiamet-G is a potent (in vitro K_i_ = 2.1 nM, cell-based EC_50_ = 21 nM) stable mimic of the oxazoline-like transition state used by OGA active site during the OGA-catalyzed hydrolysis of O-GlcNAc, leading to an increase of O-GlcNAc modification of proteins. Within cells, the prodrug Ac_4_5SGlcNAc generates the OGT inhibitor UDP-5SGlcNAc (in vitro K_i_ = 5 µM) and decreases cellular O-GlcNAcylation (cell-based EC_50_ = 5 µM). Both compounds have been evaluated in SK-N-AS cells for imaging the changes of tau *O*-GlcNAc [12], but to the best of our knowledge, this is the first study to compare the inhibitory effect of thiamet-G and Ac_4_5SGlcNAc upon *O*-GlcNAcylation on β-catenin in living cells (Figure 6).

When GFP-3a-β-catenin-transfected HeLa cells were treated with 1 µM thiamet-G 2 h before or after incubation with 200 µM Ac_4_GalNAz, and finally labeled with DBCO-cy3, a decrease of the pb-FRET signal was recorded consistent with a higher rate of occupancy of O-GlcNAc moieties on β-catenin in response to OGA inhibition (Figure 6).

Conversely, when GFP-3a-β-catenin-transfected HeLa cells were incubated with 100 µM Ac_4_5SGlcNAc for 2 h prior to the treatment with Ac_4_GalNAz and labeled with DBCO-cy3, a large increase (2.1%; see Figure 6) of the pb-FRET signal was recorded compared to the untreated cells. This observation was consistent with previous studies [16,29] that showed a major reduction in cellular *O*-GlcNAc when using this inhibitor. Indeed, the expression of OGT increased in response to its inhibition [30,31,32]. Thus, the blockade of its activity by UDP-5SGlcNAc resulted, as expected, in higher OGT expression, as previously demonstrated and also seen with the OGT inhibitor OSMI-1 [30] or upon glucose deprivation [31,32]. It should also be noted here that the substrate was present in an excess amount, compared to the inhibitor in our step-up, as Ac_4_GalNAz concentration was higher than Ac_4_5SGlcNAc concentration, and effective inhibition nevertheless still occurred. On the other hand, adding the OGT inhibitor after treating the cells with Ac_4_GalNAz had no effect, with the same pb-FRET signal being recorded as that of the control cells incubated only with the azido-sugar. The absence of an increase in signal was most likely due to the fact that the incubation time with the inhibitor was too short to observe an increase in the expression of OGT, as we assumed for the previous condition.

## 3. Discussion

The Human Genome Project consortium revealed that human DNA coded for only a third of the 100,000 genes expected [33]. Since then, this number is continuing to be lowered with a current estimate of about 21,000 genes. Yet, the human proteome is very broad since it is estimated to be capable of regulating more than 500 million different biological activities. In addition to the mechanisms of alternative splicing of the transcribed primary proteins, post-translational modifications increase exponentially the number of possible protein isoforms [4]. These PTMs alter proteins functions through various mechanisms, such as changes in protein-protein interactions, altered stability, or adaptation of catalytic activity to the environmental context. Owing to the vast diversity and the complexity of PTMs, studying them is usually challenging; the difficulty comes with the specific tagging of one precise form of the protein of interest and the lack of imaging tools to permit tracking of this protein form [3,7,11,13]. In this regard, the FRET technologies described in this work may help with visualizing the glycosylation state of specific glycoproteins and also provide the bases for characterizing glycomes with molecular precision.

Our present work refers to the specific *O*-GlcNAcylation PTM that occurs mostly intracellularly. Indeed, *O*-GlcNAcylation regulates a large set of biological functions in a nutrient-dependent manner [4,34]. Dysregulation of *O*-GlcNAcylation processes is observed in a variety of diseases, including neurodegenerative diseases, metabolic disorders, cardiovascular disorders, and cancers.

A better understanding of the *O*-GlcNAcylation function, protein by protein, would certainly translate in significant progress in the knowledge and treatment of these diseases. However, there are few existing molecular tools that allow a rapid and easy determination of the *O*-GlcNAcylation status of a protein of interest in live cells, rendering such progress difficult to make.

**Figure 7:**
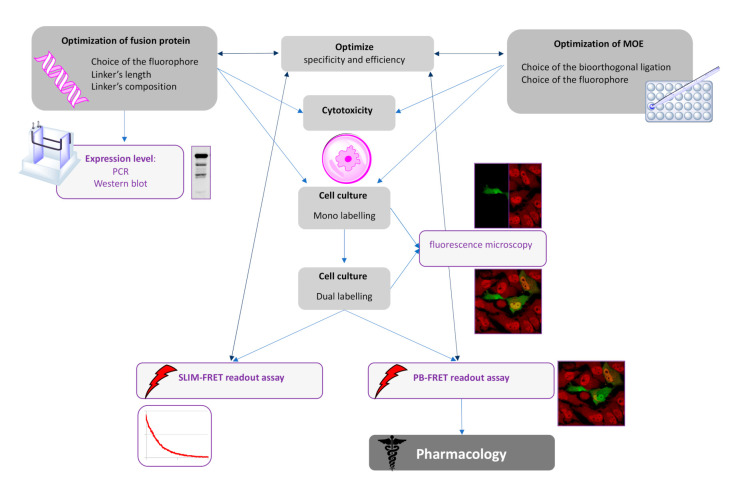
Flowchart demonstrating how to combine the technologies for FRET-based imaging of dual-labeling of glycoproteins.

In this study, we successfully adapted the dual-labeling method initially developed by Lin and co-workers [13] to detect a β-catenin glycoform using FRET techniques. As the selection of a suitable linker of GFP-tagged proteins is often neglected and underexplored, we focused a part of our study in the design and the choice of the linker between GFP and β-catenin. As FRET is contingent upon the ability to precisely introduce a suitable pair of donor and acceptor fluorophores into the protein of interest, we concomitantly optimized not only the linker but also the chemical reporter concentration. We validated our approach using a combination of azido sugar labeling and two-photon fluorescence lifetime imaging spectroscopy (SLiM). As a negative control, we also used an alkyne sugar analog (which is unable to react with the selected fluorophore via a SPAAC reaction) to confirm intramolecular FRET. Thereafter, we turned our attention to demonstrate the robustness of our technology using a conventional confocal microscope to monitor FRET through a pb-FRET readout assay. With this technology in our hands, we evaluated changes of β-catenin O-GlcNAcylation by inhibiting OGA or OGT. Finally, we provided a flowchart demonstrating how these technologies could be meaningfully combined (Figure 7).

We expect that the proof of concept established by our study will serve as a general and useful approach to studying the role of *O*-GlcNAcylation on specific protein targets. Our strategy permits a more precise way of studying these effects compared with pharmacological inhibition, which results in global perturbations of *O*-GlcNAc levels. Additionally, our approach using β-catenin as an acceptor protein represents an effective cell-based reporter assay of OGT activity, which could be further optimized for a variety of applications. For example, we believe that our methodology could be adapted for future studies to screen for inhibitors of OGT or OGA.

This methodology, developed on a precise cell system, protein, and PTM, should open up interesting perspectives in various fields of cell biology. This system should be fairly easily adaptable to any type of glycosylation, given that MOE is an established technology with a broad scope of sugars that can be incorporated in vitro. We also expect that in the future, this strategy will be useful for the study of other PTMs after the requisite optimization towards a particular type of modification. In view of the speed of coordination between chemists and biologists, such tools should soon emerge for many modifications other than glycosylations.

## 4. Materials and Methods

### 4.1. Chemical Compounds

Ac_4_GalNAz, Ac_4_GAlNAl, and DBCO-Cy3 were purchased from Click Chemistry Tools (Scottsdale, USA). Thiamet-G and Ac_4_5SGlcNAc were prepared, as previously described [26,30].

The tetraacetylated *N*-azidoacetyl-mannosamine (Ac_4_ManNAz) was synthesized from ManNAz. The crude product was purified by silica column chromatography with Cyclohexane/Ethyl acetate (2:1 *v*/*v*) and characterized by NMR ^1^H, COSY, and HSQC experiments (yield 88%). The obtained white powder corresponded to a pure mixture of non-assessed α/β anomers (60/40).

^1^H NMR (300 MHz, CDCl_3_) δ = 6.64 (d, J = 8.9, 1H’), 6.56 (d, J = 9.2, 1H), 6.05 (d, J = 1.9, 1H), 5.89 (d, J = 1.6, 1H’), 5.34 (dt, J = 11.7, 5.9, 1H), 5.27–5.12 (m, 1H + 1H’), 5.10−5.00 (m, 1H’), 4.73 (ddd, J = 9.0, 3.8, 1.6, 1H’), 4.62 (ddd, J = 9.3, 4.2, 1.9, 1H), 4.25 (dt, J = 12.4, 4.4, 1H + 1H’), 4.19−3.99 (m, 4H + 3H’), 3.82 (ddd, J = 9.6, 4.5, 2.5, 1H’), 2.19 (s, 3H + 3H’), 2.12 (s, 3H + 3H’), 2.05 (s, 3H + 3H’), 2.01 (s, 3H + 3H’).

### 4.2. Cell Culture

HeLa cells were obtained from the American Tissue Culture Collection. Cells were grown in Dulbecco’s modified Eagle’s medium (Lonza) supplemented with 10% (*v*/*v*) of fetal-calf serum (Lonza). Cells were maintained at 37 °C in a humidified atmosphere containing 5% (*v*/*v*) CO_2_.

### 4.3. Cell Toxicity

HeLa were seeded in 96-well plate (15,000 cells/well) in 100 µL DMEM. After 24 h, the medium was replaced with DMEM containing Ac_4_GalNAz at various concentrations (0, 10, 20, 50, 100, 200, 300, 400, 500, 600, 800, and 1000 µM). Each condition was replicated 6 times. Twenty-four hours later, MTS assay was performed: 20µL of MTS/PMS solution was added in each well, and cells were incubated for 2 h at 37 °C/5% (*v*/*v*) CO_2_. The absorbance was then read at 490 nm using a microplate reader.

### 4.4. Plasmids

vCMVp-GFP-β-catenin, vCMVp-GFP-1a-β-catenin, vCMVp-GFP-2a-β-catenin, vCMVp-GFP-3a-β-catenin, vCMVp-GFP-3A-β-catenin-CFP-β-catenin were generated by e-Zyvec (www.e-zyvec.com, Loos, France). Linkers between GFP and β-catenin were an alanine-rich amino acid sequence repeated one, two, or three times: 5′-EAAAKEAAAKEAAAKEAAAKA-3′.

### 4.5. Transfections

For microscopy, cells were grown on glass coverslips and transfected at 70% confluency. Transfections were performed using 1 µL of Jetoptimus (Polyplus), 1 µg of the plasmid in 900 µL DMEM, according to the manufacturer’s instruction. The transfections mix was replaced 4 h later with fresh medium containing Ac_4_GAlNAz, Ac_4_GalNAl, or Ac_4_ManNAz (200 µM), with or without Ac_4_5SGlcNAc (100 µM) or Thiamet G (1 µM), for 24 h.

### 4.6. Western Blot Analyses

Cells were washed once in ice-cold PBS and lysed in RIPA buffer (Tris/HCL 50mM, NaCl 150 mM, NP40 0,5% (*v*/*v*), EDTA 1 mM, Na_3_VO_4_ 1mM, NaF 5 mM, pH 7.9) supplemented with a protease inhibitors mix (Roche Diagnostics) for 20 min on ice. The lysate was centrifuged at 5000 rpm, 4 °C for 10 min, and the supernatants were collected. Protein quantitation was determined by using the BCA assay (ThermoFischer). Equal quantities (15–20 µg) of proteins were loaded and separated by 10% SDS-PAGE. Proteins were transferred onto a nitrocellulose membrane (GE Healthcare) at 200 mA for 2 h. Membranes were blocked for 1 h at room temperature with 5% (*w*/*v*) nonfat dry milk in Tris-buffered saline (TBS) Tween (TBST) buffer (15 mM Tris, 140 mM NaCl, 0.05% (*v*/*v*) Tween20, pH 8.0). Membranes were incubated overnight at 4 °C with anti-β-catenin (1:2000) (H102, Santa Cruz Biotechnology, Santa Cruz, CA, USA) or anti-GFP (1:1000) monoclonal antibodies. After three washes with TBST, membranes were incubated with corresponding secondary HRP-linked antibodies (1:10,000 in TBST) for 1 h at room temperature. After three washes in TBST, the detection was performed with a CCD camera (Fusion Solo, Vilber Lourmat, Marne-la-Vallee, France).

### 4.7. O-GlcNAc Mass Tag Labeling

The experimental procedure we followed was previously described by Leturcq et al. 2018 [34]. Briefly, HeLa cells were transfected with the different GFP-β-catenin constructs (Figure 2a) and treated with 200 µM of Ac_4_GalNAz for 24 h. Then, 50 µg of the labeled proteins were resuspended in 20 mM HEPES and 1% (*w*/*v*) SDS, pH 7.9, and incubated for 1 h at room temperature with a 4.4 kDa DBCO-PEG (polyethylene glycol) at a final concentration of 10 mM in DMSO. DBCO-PEG was synthesized, as previously described [35]. One volume of PEGylated proteins was precipitated using the mix of chloroform:methanol:water (3:0.75:2) in order to remove the excess of DBCO-PEG. Proteins were heated at 95 °C for 10 min, resolved by SDS-PAGE, and analyzed by Western blot.

### 4.8. Sample Preparation and Bioorthogonal Ligation for Fluorescence Microscopy

Cells cultured on coverslips were washed three times in Dulbecco’s Phosphate Buffer Saline (Lonza) containing calcium and magnesium. Cells were fixed with 4% (*w*/*v*) paraformaldehyde (pH 7.3) for 30 min at room temperature and washed with ice-cold PBS thrice. Coverslips were switched in a humid chamber. For SPAAC, 10 µM DBCO-Cy3 was applied for 1 h. Cells were washed three times with ice-cold PBS, and the coverslips were mounted in mounting medium (Dako).

### 4.9. Spectral Fluorescence Lifetime Imaging Microscopy

Lifetime measurements were performed using an MW-FLIM detector and an SPC 150 photocounting card from Becker & Hickl (Becker & Hickl, Berlin, Germany) adapted on a laser scanning microscope LSM 710 NLO Zeiss (Zeiss SAS, Jena, Germany) and coupled with a Chameleon TiSa accordable 80 MHz pulsed laser (COHERENT, Santa Clara, USA). For more details on the acquisition and analysis procedure for FRET, please see [24]. For GFP lifetime measurements, acquisitions were performed at 860 nm and detection between 500 and 560 nm. Lifetime values were extracted from a minimum of 10 cells per experiment.

### 4.10. Photobleaching FRET

The samples were observed under the A1 Nikon confocal microscope with a 60X oil immersion objective. The green fluorescence (GFP) was acquired with λex = 488 nm and λ_em_ = 500–530 nm, and the red fluorescence (Cy3) was acquired with λex = 561.6 nm and λ_em_ =570–620 nm. Photobleaching of the acceptor was achieved by increasing laser power from 10% to 100%, increasing the zoom from 1 to 16, and by scanning the selected area 10 times. Images acquired before and after photobleaching were processed, according to [36], with ImageJ. Photobleaching FRET values were extracted from a minimum of 10 cells per experiment.
